# An Improved Synthesis of N-(4-[^18^F]Fluorobenzoyl)-Interleukin-2 for the Preclinical PET Imaging of Tumour-Infiltrating T-cells in CT26 and MC38 Colon Cancer Models

**DOI:** 10.3390/molecules26061728

**Published:** 2021-03-19

**Authors:** Shivashankar Khanapur, Fui Fong Yong, Siddesh V. Hartimath, Lingfan Jiang, Boominathan Ramasamy, Peter Cheng, Pradeep Narayanaswamy, Julian L. Goggi, Edward George Robins

**Affiliations:** 1 Singapore Bioimaging Consortium, Agency for Science, Technology and Research (A* STAR), 11 Biopolis Way, #01-02 Helios, Singapore 138667, Singapore; Yong_Fui_Fong@sbic.a-star.edu.sg (F.F.Y.); S_Hartimath@sbic.a-star.edu.sg (S.V.H.); Jiang_Lingfan@gis.a-star.edu.sg (L.J.); Boominathan_Ramasamy@sbic.a-star.edu.sg (B.R.); Peter_Cheng@sbic.a-star.edu.sg (P.C.); Julian_Goggi@sbic.a-star.edu.sg (J.L.G.); 2 Sciex, R&D, Blk 33, #04-06 Marsiling Industrial Estate Road 3 Woodlands Central Industrial Estate, Singapore 739256, Singapore; pradeep.naraya@sciex.com; 3 Clinical Imaging Research Centre, 14 Medical Drive, #B01-01 Centre for Translational Medicine, Yong Loo Lin School of Medicine, National University of Singapore, Singapore 117599, Singapore

**Keywords:** positron emission tomography (PET) imaging, Interleukin-2 (IL-2), T-cells, [^18^F]FB-IL-2, [^18^F]SFB, Scintomics GRP™ module, protein conjugation reaction, murine colon adenocarcinoma (MC38 and CT26) syngeneic models

## Abstract

Positron emission tomography (PET) imaging of activated T-cells with *N*-(4-[^18^F]fluorobenzoyl)-interleukin-2 ([^18^F]FB-IL-2) may be a promising tool for patient management to aid in the assessment of clinical responses to immune therapeutics. Unfortunately, existing radiosynthetic methods are very low yielding due to complex and time-consuming chemical processes. Herein, we report an improved method for the synthesis of [^18^F]FB-IL-2, which reduces synthesis time and improves radiochemical yield. With this optimized approach, [^18^F]FB-IL-2 was prepared with a non-decay-corrected radiochemical yield of 3.8 ± 0.7% from [^18^F]fluoride, 3.8 times higher than previously reported methods. In vitro experiments showed that the radiotracer was stable with good radiochemical purity (>95%), confirmed its identity and showed preferential binding to activated mouse peripheral blood mononuclear cells. Dynamic PET imaging and ex vivo biodistribution studies in naïve Balb/c mice showed organ distribution and kinetics comparable to earlier published data on [^18^F]FB-IL-2. Significant improvements in the radiochemical manufacture of [^18^F]FB-IL-2 facilitates access to this promising PET imaging radiopharmaceutical, which may, in turn, provide useful insights into different tumour phenotypes and a greater understanding of the cellular nature and differential immune microenvironments that are critical to understand and develop new treatments for cancers.

## 1. Introduction

Cancer immunotherapy (CIT), the fourth pillar of cancer therapy, utilizes the body’s own immune system to fight cancer. Recently, CIT has garnered significant interest with the approval of several immune checkpoint inhibitors (Programmed cell death protein 1, Programmed death-ligand 1 and cytotoxic T-lymphocyte-associated protein 4), cancer vaccines and adjuvants (Dendritic cell vaccines and CpG, tumour protein), cytokine therapies (Interferon alfa , IL-2) and adaptive cell-based immunotherapies (Chimeric antigen receptor T-cells, Tumour-infiltrating T-cells, Natural killer cells) [1]. Despite the success of CIT across a broad range of human cancers, only a minority of patients show a durable response to these therapies and some patients experience severe immune-related adverse effects. Therefore, there is a tremendous need to stratify patients and optimize immunotherapy combinations for better management of cancer patient care [2]. Immune responses are commonly assessed by measuring lymphocytes in whole blood and/or by biopsies of tumour, spleen and lymph nodes. These methods are invasive and do not provide spatiotemporal information about the dynamic immune responses to these therapies in heterogeneous tumour microenvironments. In this context, non-invasive positron emission tomography (PET) imaging of immune cells, especially T cells, might aid in patient selection, response prediction and treatment evaluation following immunotherapeutic intervention [1].

T-cells are crucial regulators of adaptive immune responses in both immune-driven diseases and tumours [3]. Upon activation by antigen-presenting cells, T-cells (CD4+ and CD8+ T-cells) secrete the protein interleukin-2 (IL-2) to regulate immune responses. IL-2, a small monomeric glycoprotein (15.3 kDa) lymphokine, binds with high affinity to its cell surface interleukin-2 receptors (IL-2Rs), which consists of three receptor subunits: α (CD25), β (CD122) and γ (CD132). Over-expression of IL-2Rs is found mainly on the activated T cells [4,5]. Radiolabelling of human recombinant IL-2 with short-lived positron-emitting radionuclides such as fluorine-18 (^18^F^−^) have already been shown to provide a tool for non-invasive imaging of activated tumour infiltrating lymphocytes (TILs) in cancer.

Several radiopharmaceuticals for PET imaging of IL-2Rs on activated T-cells have already been developed [5,6,7,8]. Perhaps the most well-characterized of these is *N*-(4-[^18^F]fluorobenzoyl)-interleukin-2 ([^18^F]FB-IL-2), which was developed as a tool for the in vivo identification and quantification of CD25+ T-cells and is currently being evaluated in clinical trials (NCT02922283 and NCT03304223) [5,6,7,9,10]. However, due to a complex production process and low radiochemical yields (RCYs) from protein conjugation reactions, the [^18^F]FB-IL-2 synthesis requires a high initial amount of radioactivity to provide a suitable quantity of formulated [^18^F]FB-IL-2 radiopharmaceutical for either clinical or preclinical use [6,7].

Therefore, we sought to optimize the synthetic steps to improve RCY and reduce the synthesis time described in existing literature radiosynthesis methods for [^18^F]FB-IL-2. In this article, we describe an improved semi-automated synthesis of [^18^F]FB-IL-2 via *N*-succinimidyl 4-[^18^F]fluorobenzoate ([^18^F]SFB) and compare it with previous manufacturing procedures. Furthermore, we characterized the synthesized radiotracer in vitro and in vivo and assessed the efficacy of [^18^F]FB-IL-2 as an imaging biomarker in preclinical PET imaging of CD25+ TILs in syngeneic murine colon cancer models.

## 2. Results

### 2.1. Radiochemistry

**Scheme 1:**
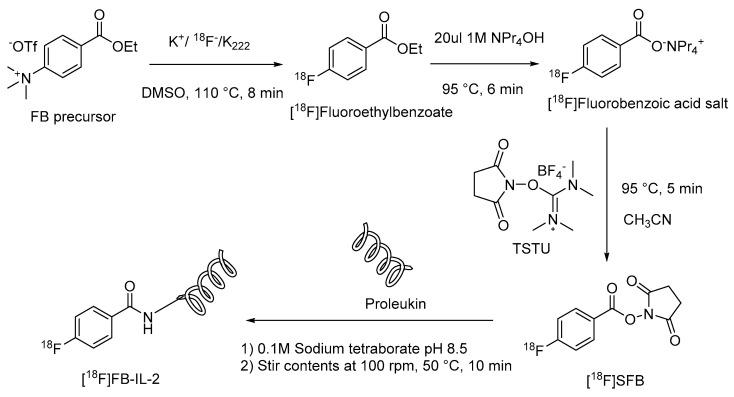
Semi-automated synthesis of [^18^F]FB-IL-2.

We have adapted the four-step, two-pot procedure to synthesize [^18^F]FB-IL-2 from those previously reported with several modifications as shown in Scheme 1 [5,7,10,11]. In this work, crude [^18^F]SFB was synthesised via three-step reactions (Scheme 1) using a Scintomics GRP™ module. Manual purification of crude reaction mixture by semi-preparative reverse phase-high performance liquid chromatography (RP-HPLC) followed by solid-phase extraction (SPE) procedure delivered pure [^18^F]SFB in a final volume of 1 mL diethyl ether which was evaporated to dryness for the downstream application. Semi-automated synthesis produced dried [^18^F]SFB with a good non-decay corrected RCY of 22.7 ± 3.5% (3.6 ± 0.9 GBq) from aqueous fluoride activity (15.9 ± 2.8 GBq) in 85 ± 7 min (n = 15). The radiochemical purity (RCP) of the [^18^F]SFB after SPE process was > 95% (Figure S2) and molar activities were >100 GBq/µmol (Supplementary Materials).

**Figure 1:**
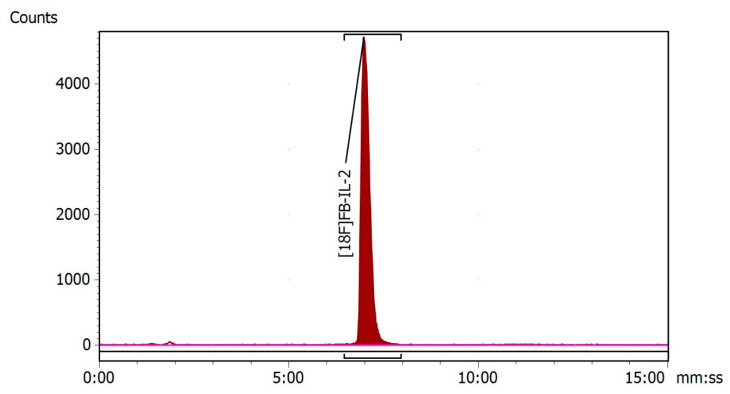

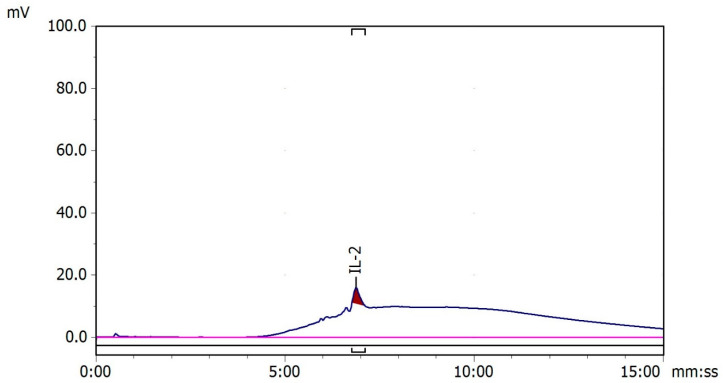
Radio- and UV chromatograms of the PD-10 purified formulated radiotracer, [^18^F]FB-IL-2.

[^18^F]SFB was reconstituted in DMSO and incubated with proleukin protein in 0.1 M sodium borate buffer for 10 min at 50 °C. The [^18^F]fluoroacylation reaction produced [^18^F]FB-IL-2 with a non-decay corrected RCY of 3.8 ± 0.7% (0.6 ± 0.1 GBq) from aqueous [^18^F]fluoride activity (15.9 ± 2.8 GBq) in 115 ± 5 min (*n* = 12). Our manual radiosynthesis procedure mainly differs from previously reported protein conjugation methods in the sequence of [^18^F]SFB addition to protein-buffer mixture and the use of co-solvent dimethyl sulfoxide (DMSO) in a reduced reaction volume [5,6,7]. The RCP of the formulated fraction was >95%, as determined by RP-HPLC (Figure 1) and TCA precipitation assay. Molar activities of the formulated fractions were 114 ± 94 GBq/µmol (*n* = 6) at the end of the synthesis (Supplementary Materials). The identities of the [^18^F]SFB and [^18^F]FB-IL-2 were confirmed by spiking with reference standard SFB and recombinant IL-2 protein, respectively, in RP-HPLC.

### 2.2. Chemical Characterisation of [^18^F]FB-IL-2

**Figure 2:**
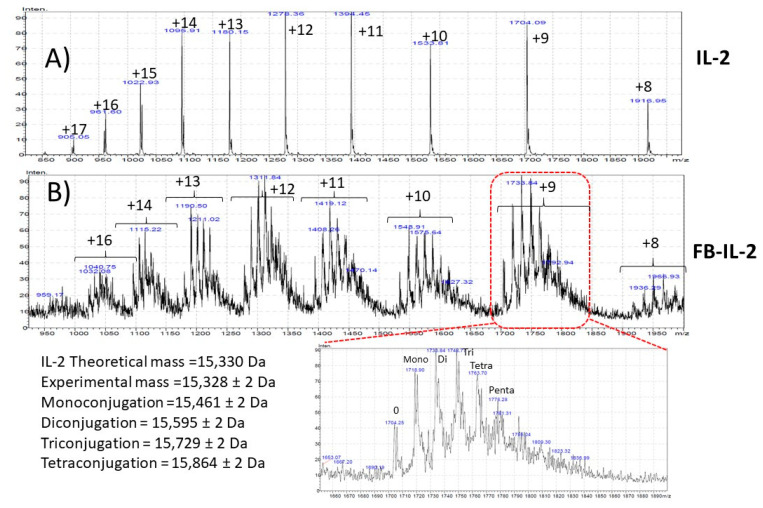
The electrospray ionization-mass spectrometry (ESI-MS) profile of unlabeled IL-2 and FB-IL-2. (**A**) The positive ion mode ESI-MS spectra of IL-2 unlabeled, showing different mass/charge (*m*/*z*) ratios from +8 to +17 charges; the deconvoluted mass of unlabeled IL-2 was 15,328 ± 2 Da and that matched the theoretical expected mass 15,330 Da. (**B**) Positive ion mode ESI-MS spectra of decayed labelled FB-IL-2 suggest a different degree of conjugation; magnified charge state (+9 charge) region suggests IL-2 labelling with monoconjugation, diconjugation, triconjugation, tetraconjugation and pentaconjugation. Based on the relative intensity ratio, it indicates between 2–3 FB groups per IL-2 molecule are the most abundant coupling. Note: The labels on the mass spectra peaks +n, indicate the number of protons attached to the protein.

The shelf-life of formulated [^18^F]FB-IL-2 solution was assessed over 5.5 h at room temperature, during which no change in RCP was observed. Liquid chromatography–mass spectrometry (LC-MS) was performed to confirm the molecular mass of the decayed labelled protein product. It was found that mass of 15,328 (unlabelled IL-2; theoretical expected mass 15,330 Da), 15,461 (monoconjugation), 15,595 (diconjugation), 15,729 (triconjugation), 15,864 (tetraconjugation) ± 2 Da for labelled FB-IL-2. LC-MS data suggest that each labelled IL-2 was coupled with 1–4 fluorobenzoyl (FB) residues with the most abundant coupling being between 2–3 FB groups (Figure 2).

### 2.3. In Vitro Biological Characterisation of [^18^F]FB-IL-2

**Figure 3:**
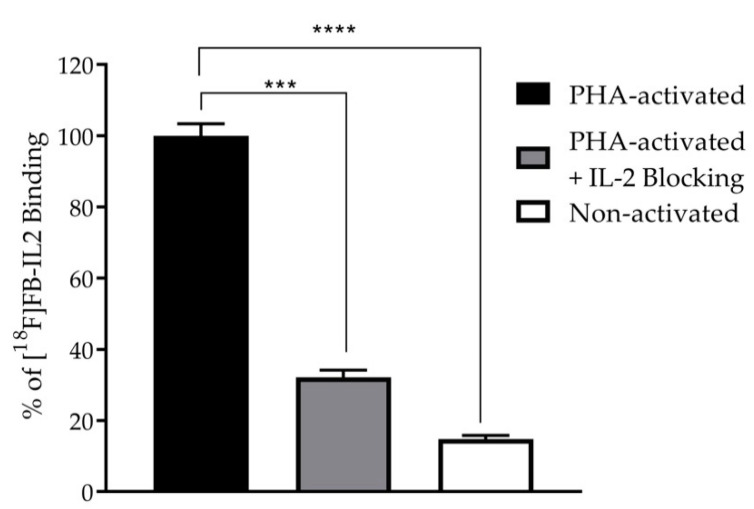
In vitro binding assessment of [^18^F]FB-IL-2 in mouse PBMCs. Selective binding of [^18^F]FB-IL-2 to PHA activated mPBMCs was observed when compared to non-activated mPBMCs (14.8 ± 0.65%, **** *p* < 0.0001). The binding was shown to be specific by a significant reduction in the binding of [^18^F]FB-IL-2 when pre-treated with excess non-radioactive recombinant IL-2 (32.12 ± 1.90, *** *p* < 0.001), data shown as % bound ± SD.

In order to assess the specificity of [^18^F]FB-IL-2 binding to IL-2Rs, in vitro experiments were performed using mouse peripheral blood mononuclear cells (mPBMCs). Phytohemoagglutinin (PHA) activated and non-activated mPBMCs were incubated with [^18^F]FB-IL-2 at 37 °C for 30 min. A significant difference in [^18^F]FB-IL-2 binding between non-activated (14.78 ± 0.65%) and activated mPBMCs (98.00 ± 2.00% *p* < 0.0001) was observed (Figure 3). In order to determine the specificity of binding, we pre-incubated with excess recombinant IL-2 protein (20 ng/mL) to saturate the IL-2Rs, which led to a threefold reduction in [^18^F]FB-IL-2 binding (32.12 ± 1.90%, *p* < 0.001).

### 2.4. Animal Studies

**Figure 4:**
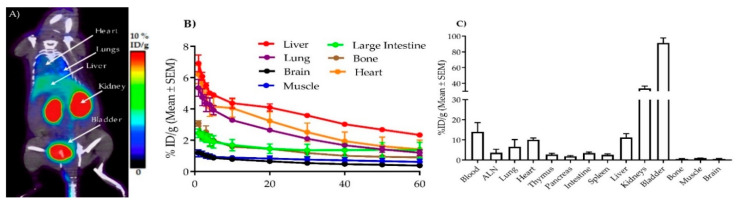
In vivo PET imaging of [^18^F]FB-IL-2 in naïve Balb/c mice. (**A**) Whole-body PET-CT fused image represents the summed frames from 40–60 min post-injection. (**B**) Dynamic PET time–activity curves showing initial uptake followed by washout of [^18^F]FB-IL-2 tracer in most of the major organs. (**C**) Ex vivo biodistribution data (70 min post-injection) showing [^18^F]FB-IL-2 tracer accumulation in different organs confirming PET-based biodistribution.

In vivo PET imaging with [^18^F]FB-IL-2 was performed in naïve mice to study tracer kinetics and tissue distribution. As shown in Figure 4A, uptake of the radiotracer could be visualized in major excretory organs, including liver and kidneys. Time–activity curves reveal a rapid uptake of the radiotracer into the liver, lungs, heart and kidney followed by exponential washout. Low radiotracer uptake was observed in the brain as the radiolabelled protein does not penetrate the blood–brain barrier (Figure 4B). Ex vivo biodistribution, Figure 4C, showed a similar overall profile, the [^18^F]FB-IL-2 radiotracer was excreted mainly via the kidneys into urinary bladder, similar to the native IL-2 and human recombinant IL-2 proteins and uptake in bone was observed to be low, indicating the lack of defluorination in vivo.

**Figure 5:**
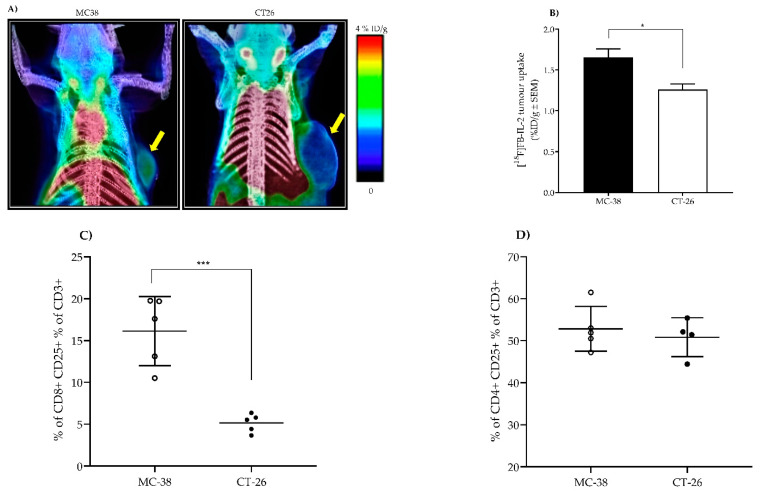
In vivo PET imaging of [^18^F]FB-IL-2 in MC38 and CT26 tumour-bearing mice. (**A**) PET-CT fused image of mice with MC38 and CT26 tumours showing the distribution of [^18^F]FB-IL-2. (**B**) Quantitative estimation of [^18^F]FB-IL-2 uptake in MC38 tumours shows significantly higher uptake than in CT26 tumours (1.65 ± 0.24 vs. 1.21 ± 0.03, data shown as mean ± SEM % ID/g, * *p* < 0.05). (**C**) FACS analysis showing CD8+CD25+ cells as a % of CD3+ cells in MC38 and CT26 tumours. CD8+CD25+ cells were significantly higher in MC38 when compared to CT26 (16.12 ± 4.14 vs. 5.14 ± 1.09, *** *p* < 0.002, data shown as mean ± SD as a % of CD3+ cells). (**D**) FACS analysis showing CD4+CD25+ as a % of CD3+ cells in MC38 and CT26 tumours. No significant differences in CD4+CD25+ cells were observed between MC38 and CT26 (data shown as mean ± SD as a % of CD3+ cells).

To investigate the utility of [^18^F]FB-IL-2 in oncology imaging, CT26 and MC38 tumour-bearing mice were imaged after 60 min of tracer injection demonstrating homogenous tumour uptake in viable tumour tissue (Figure 5A). Significantly higher uptake of [^18^F]FB-IL-2 was observed in MC38 tumours compared to CT26 tumours (1.65 ± 0.24 vs. 1.21 ± 0.03, % ID/g, *p* < 0.05, Figure 5B). Fluorescence activated cell sorting (FACS) analysis was performed on these excised colon tumours and showed that these MC38 tumours had greater infiltration of CD8+ CD25+ cells compared to CT26 tumours (16.12 ± 4.14 vs. 5.14 ± 1.09, *p* < 0.002, Figure 5C), whereas no significant difference was observed for CD4+ CD25+ cells (52.82 ± 5.31 vs. 50. 82 ± 4.62, *p* = 0.566, Figure 5D). These data suggest that FB-IL2 may be able to differentiate tumours based on their levels of CD25+ TILs.

## 3. Discussion

Here we describe an improved method of synthesis for a promising radiopharmaceutical, [^18^F]FB-IL-2, to image-activated T-cells. Semi-automated [^18^F]SFB synthesis was performed using a Scintomics GRP™ module. Crude product obtained from Scintomics was then purified using semi-preparative RP-HPLC, followed by a SPE cartridge to obtain a chemically pure intermediate radiolabelling synthon, [^18^F]SFB. Purification by RP-HPLC was adopted as it was reported earlier that inefficient [^18^F]SFB purification only by SPE cartridge resulted in low protein-conjugation reaction yields and frequent failures [7].

Dropwise addition of [^18^F]SFB was chosen to mitigate the effects of hydrolysis. In our experience, [^18^F]SFB in DMSO showed better hydrolytic stability, higher reactivity and better solubility than polar protic solvent, ethanol. In addition, the minimal reaction volume (300 µL) with the use of 1 mL V-vial and stirring the contents at 100 rotations per minute (rpm) provided higher RCYs than existing procedures [6,7].

In contrast to RP-SPE purification [7], the SEC column method of purification provided the labelled protein product with high recovery (90–95%) and without the need for an additional reformulation step. The use of SEC-PD10 column purification and elution buffer 0.05% sodium dodecyl sulphate (SDS) in phosphate-buffered saline (PBS) allowed us to streamline the purification and reformulation of the labelled product for injection into animals. [^18^F]FB-IL-2 was prepared with 3.8 times higher RCYs and 30 min less reaction time than published articles procedures (1.0 ± 0.4% in 150 min) [6,7].

No change on RCP of [^18^F]FB-IL-2 formulated product due to chemically or radiochemically induced decay was found at least for 5.5 h at room temperature, as determined by RP-HPLC in a shelf-life experiment. Additionally, LC-MS data suggest that after labeling, each labelled IL-2 was coupled with between 1 and 4 FB residues. The degree of labelling (DOL) can be determined on the basis of radioactivity incorporated in the protein (GBq), the molar activity of [^18^F]SFB (GBq/µmol), and the concentration of protein recovered after reaction (mg/mL) [5]. With this calculation, we determined that on average, 1.9–3.9 FB residues to each labelled protein molecule. The calculated value range is in agreement with our LC-MS data. In vitro radioligand binding assay showed preferential binding to activated mPBMCs and binding was selective, as determined by receptor saturation binding experiment.

In vivo [^18^F]FB-IL-2 dynamic PET imaging and ex vivo biodistribution studies showed that the formulated product has comparable tissue distribution and kinetics to previously published data [5,6,8,9]. Significantly higher uptake of [^18^F]FB-IL-2 was observed in MC38 tumours compared to CT26 tumours (Figure 5B), potentially due to phenotypic differences in the tumour types, as MC38 tumours are associated with a microsatellite instability-high (MSI-high) phenotype compared to the MSI-low CT26 tumours. Typically, high MSI phenotype tumours have high mutation rates due to DNA mismatch repair and increased immunogenicity of neoantigens. As a result, MSI-high phenotype tumours tend to be more immunologically active with higher numbers of CD25+ TILs.

## 4. Materials and Methods

### 4.1. General Information

The compounds 4-(ethoxycarbonyl)-*N,N,N*-trimethylbenzenaminium trifluoromethanesulfonate (FB precursor) and 2,5-dioxopyrrolidin-1-yl 4-fluorobenzoate (reference standard) were procured from ABX GmbH, Radeberg, Germany. Proleukin^®^ (aldesleukin, 18 × 10^6^ IU), a recombinant interleukin-2 (desalanyl-1, serine-125 human interleukin-2), was acquired from Novartis, Singapore. *N,N,N′,N′*-Tetramethyl-*O*-(*N*-succinimidyl)uronium tetrafluoroborate (TSTU, 97%), acetonitrile anhydrous (99.8%), potassium carbonate anhydrous (99.99%), 4,7,13,16,21,24-hexaoxa-1,10-diazabicyclo[8.8.8]hexacosane (Kryptofix^®^222, 98%), dimethyl sulfoxide anhydrous (≥99.9%), tetrapropylammonium hydroxide solution (TPAOH, 1.0 M in H_2_O), sodium dodecyl sulfate (SDS, ≥99.0%) and sodium tetraborate decahydrate (≥99.5%) were procured from Sigma-Aldrich Pte Ltd., Singapore. All other reagents were procured from Merck (Singapore), Tokyo Chemical Industry and Life Technologies Corporation (Singapore). All commercially obtained reactants and reagents were used as received without any further purification. [^18^F]SFB and [^18^F]FB-IL-2 productions were carried out in a closed type-I glass flat bottom vial (15 mL) and closed Thermo Scientific™ conical Reacti-vial™ (1 mL), respectively. Sep-Pak^®^ light (46 mg) Accell™, QMA carbonate, Oasis HLB and light cartridges were purchased from Waters Pacific Pte Ltd., Singapore. PD-10 desalting columns were obtained from GE Healthcare Life Sciences, Singapore.

No-carrier-added (nca) aqueous [^18^F]fluoride ion was produced by the irradiation of ^18^O-enriched water via the [^18^O(p,n)^18^F] nuclear reaction using a GE PETtrace 860 cyclotron (GE healthcare, Singapore). Radiochemical purification and radio-UV HPLC analyses were performed on a Knaur semi-preparative and UFLC Shimadzu radio-HPLC systems (Shimadzu, Singapore), respectively [2]. Radioactivity measurements were made with a CRC-55tPET dose calibrator (Capintec, Florham Park, NJ, USA).

### 4.2. [^18^F]FB-IL-2 Radiosynthesis via Semi-Automated Synthesis of [^18^F]SFB

IL-2 was labelled with fluorine-18 by conjugation of the Proleukin protein (Novartis, Singapore) with *N*-succinimidyl 4-[^18^F]-fluorobenzoate ([^18^F]SFB). Scintomics GRP4V cassette module was used to perform synthesis of crude [^18^F]SFB. Details on the setup and connections of the tubing are described in the supplementary materials.

At the beginning of the automated sequence, nca aqueous [^18^F]fluoride activity (obtained from cyclotron; 2.4 mL) was transferred directly under suction into a V-vial placed in the hot cell. Trapping of nca [^18^F]fluoride activity onto a pre-conditioned Sep-Pak Light Waters Accell™ Plus QMA carbonate cartridge (manual precondition was done by passing 10 mL of deionised water) took place under vacuum along with the separation of enriched water. The trapped [^18^F]fluoride anion was then eluted with a solution of 3 mg K_2_CO_3_ (21.9 µmol) and 15 mg Kryptofix^®^ 222 (39.9 µmol) in a mixture of 40 μL water and 960 µl acetonitrile into an empty reactor vial. Azeotropic drying of the [K(K_222_)]^+^ [^18^F]^−^ complex was performed under reduced pressure at 95 °C under a gentle stream of nitrogen gas (40 mL/min). After the first round of drying, anhydrous acetonitrile (0.5 mL) was added to the [K(K_222_)]^+^ [^18^F]^−^ complex and the drying step was repeated. Following drying, 5 mg FB precursor (14 μmol) in 1 mL DMSO was added to the dried complex [K(K_222_)]^+^ [^18^F]^−^.

The fluorination reaction was allowed to react for 8 min at 110 °C to provide ethyl-4-[^18^F]fluorobenozoate. It was then deprotected using 20 µL TPAOH (1.0 M in H_2_O) in 1.5 mL acetonitrile, providing the corresponding tetrapropylammonium salt. Subsequent azeotropic drying and then a coupling reaction with 25 mg TSTU (83.1 µmol) in 1.5 mL acetonitrile yielded crude [^18^F]SFB (Scheme 1). The purification of [^18^F]SFB was achieved by preparative HPLC followed by the SPE procedure as previously described [2]. After reconstitution with DMSO, analytical chromatography to determine RCP and molar activity was carried out using a Synergi™ 4 µm Fusion-RP 80 Å, LC Column 250 × 4.6 mm at a flow rate of 1.5 mL/min. Gradient elution was carried out using a mixture of 0.1% aqueous trifluoroacetic acid (solvent A) and acetonitrile (solvent B). The following gradient elution profile was used: 0.01–0.30 min 10% B, 0.30–5.00 min 95% B, 5.00–8.00 min 95% B, 8.00–12.00 min 10% B, λ = 254 nm. The retention time of [^18^F]SFB was between 7.0–7.1 min.

Dried [^18^F]SFB was reconstituted with 100 µl DMSO and added dropwise into pH 8.5 adjusted mixture of 100 µL aqueous IL-2 solution (Proleukin; 2 mg/mL) and 100 µL 0.1 M sodium borate buffer in 1 mL V-vial at 50 °C for 10 min while stirring the reaction mixture at 100 rpm (Scheme 1). The crude reaction mixture was then purified by SEC using PD-10 column, which was pre-conditioned with 30 mL elution buffer (0.05% SDS in PBS). The pure labelled [^18^F]FB-IL-2 protein was eluted in 4 fractions of 0.5 mL each.

The RCP, radiochemical identity and molar activity of the [^18^F]FB-IL-2 was determined using analytical radio-HPLC. The stationary phase was a Phenomenex Aeris Widepore C4, 3.6 μm, 150 mm × 2.1 mm column. Water with 0.1% trifluoroacetic acid (solvent A) and acetonitrile with 0.1% trifluoroacetic acid (solvent B) were used as a mobile phase. The following gradient elution profile was used: 0.2 min 5% B, 0.2–5 min 95% B, 5–6 min 95% B, 6–14 min 5% B, flow rate of 0.9 mL/min, λ = 280 nm. The retention time of [^18^F]FB-IL-2 was between 6.5–7.1 min (Figure 1). The RCP and radiolabelling efficiency of [^18^F]FB-IL-2 was determined using TCA precipitation assay. In brief, 5 µL of [^18^F]FB-IL-2 was added to 1 mL of ice-cold PBS and 5 µL of 20% human serum albumin (HSA). The protein was precipitated by adding 1 mL of 20% ice-cold TCA and then centrifuged at 4000 rpm for 5 min. A pellet was formed at the bottom and 1 mL of clear supernatant was transferred to a separate tube. The radioactivity in each fraction was counted on a gamma counter (Wizard 2470, PerkinElmer, Singapore). The percentage of labelling efficiency was determined using the formula: percentage of protein-bound activity (%) = [(activity in pellet − supernatant)/(activity in pellet + supernatant) × 100].

### 4.3. Chemical Characterization of [^18^F]FB-IL-2

The stability of the formulated [^18^F]FB-IL-2 solution was assessed longitudinally at room temperature using analytical RP-HPLC (Aeris Widepore C4, 3.6 µm, 150 mm × 2.1 mm). A gradient elution was carried out using a mixture of 0.1% aqueous trifluoroacetic acid (solvent A) and 0.1% trifluoroacetic acid in acetonitrile (solvent B), similar to analytical method parameters. Samples were taken at 0, 1 h, 2 h, 3 h, and 5 h. Experiments were performed in duplicate for each time point (*n* = 2). RCP was calculated by integration of the peak areas in the chromatogram obtained.

A LC-MS 2020 (Shimadzu Asia Pacific Pte Ltd., Singapore) was used for identification of the labelled protein. While in post-radioactive decay, a sample was subjected to LC-MS analysis using an Agilent Poroshell 120 EC-18, 2.7 µm, 4.6 × 50 mm column at a flow rate of 0.4 mL/min. Gradient elution was carried out using a mixture of 0.1% aqueous formic acid (solvent A) and 0.1% formic acid in acetonitrile (solvent B). The following gradient elution profile was used: 0.00–8.00 min 10% B, 8.00–12.00 min 95% B, 12.00–12.50 min 10% B, 12.50–15.00 min 10% B, λ = 254 nm. The mass spectrometer was operated in electrospray positive ionization mode. The mass spectrometer settings were optimized as follows: interface voltage, 4.5 kV; nebulizer gas flow, 1.5 L/min; drying gas flow, 15 L/min; desolvation line (DL) temperature, 250 °C; heat block temperature, 250 °C. Other mass spectrometer parameters were tuned automatically. Mass spectral data analysis was done manually for identification of labelled protein.

### 4.4. In Vitro Biological Characterisation of [^18^F]FB-IL-2

To assess [^18^F]FB-IL-2 binding and specificity for IL-2Rs, an in vitro binding experiment was carried out using mPBMCs. mPBMCs were isolated from fresh mouse blood by density gradient centrifugation using Lymphoprep^®^ separation (STEMCELL Technologies). Isolated mPBMCs were kept in RPMI-1640 medium supplemented with 0.05% bovine serum albumin and 1% Penicillin Streptomycin solution. Isolated cells were activated or non-activated by incubation with or without 10 μg/mL of PHA (Sigma-Aldrich, Singapore), respectively, in a 5% CO_2_ atmosphere at 37 °C for 48 h. Approximately 1 × 10^5^ cells were incubated with 300 kBq tracer solution at 37 °C for 30 min. After incubation, unbound activity was removed by washing with 1 mL ice-cold PBS and cell-bound activity was measured using a gamma counter. A receptor saturation binding experiment was performed where activated mPBMCs were pre-incubated with IL-2 (20 ng/mL) at 4 °C for 30 min, prior to addition of the [^18^F]FB-IL-2.

### 4.5. Animal Studies

All animal procedures were carried out in accordance with the Institute Animal Care and Use Committee (IACUC No. 181399) and conformed to the US National Institutes of Health (NIH) guidelines and public law. BALB/c and C57BL/6 mice of 5–7 weeks were purchased from local supplier (InVivos, Singapore) and housed in an individual ventilated cage with a 12 h day/night cycle regimen, fed standard laboratory chow and tap water ad libitum. The murine colon tumour cell lines, CT26 and MC38, were purchased from ATCC and cultured in RPMI-1640 supplemented with 10% fetal bovine serum (FBS), 100 U/mL penicillin and 100 μg/mL streptomycin, and maintained at 37 °C in a humidified incubator (with 5% CO_2_). Mice were subcutaneously implanted with CT26 or MC38 cells (1 × 10^6^) prepared in a mixture of matrigel and PBS (1:1 ratio) into the right shoulder of BALB/c or C57BL/6 mice, respectively. Tumour growth was monitored by caliper measurement and the tumour volume was calculated by using the modified ellipsoid formula ½(Length × width^2^).

To study the normal tissue distribution and kinetics of [^18^F]FB-IL-2, in vivo PET imaging was performed in naïve Balb/c mice (*n* = 5) using an Inveon PET-CT scanner (Siemens). Mice were anesthetized using a mixture of isoflurane and medical air (5% induction, 2% maintenance) and kept on electronic heating pads during the scanning period. Mice were injected with 15 ± 2 MBq of [^18^F]FB-IL-2 via the lateral tail vein and immediately a dynamic scan of 60 min was performed. During the scanning, animals were monitored for body temperature and respiration rate using a Biovet physiological monitoring system. After the scan, mice were euthanized and different organs were harvested, weighed, and the tissue-bound radioactivity was measured.

Mice bearing CT26 and MC38 colon tumours were injected with 7 ± 1 MBq via the lateral tail vein (*n* = 5 per group). A static PET acquisition of 10 min was performed after 40 min of post-tracer injection, followed by a CT scan (40 kV, 500 µA; 4 × 4 binning, 200 µm resolution) for co-registration. PET images were corrected for decay, scatter and iteratively reconstructed to 11 frames (4 × 60, 1 × 120, 2 × 300, 4 × 600 s) and 2 frames (2 × 5 min) for dynamic and static PET scans, respectively. The radioactive uptake in different organs, including the tumour, was estimated by drawing a volume of interest (VOI) delineated by CT. The VOIs were transferred from the CT template to the PET data and regional time–activity curves were generated. The image analysis of reconstructed calibrated images was performed using Amide software (version 10.3 Sourceforge) and data are expressed as a percentage of injected dose per gram (% ID/g) in the VOI.

After scanning, the mice were euthanized and tumours were harvested for flow cytometry analysis as described previously [2]. In brief, tumours were mechanically digested by incubating in 20 μg/mL DNAse1 (Sigma-Aldrich, Singapore) and 200 μg/mL collagenase (Sigma-Aldrich, Singapore) in RPMI medium supplemented with 10% heat-inactivated FBS for 1 h at 37 °C. A single-cell suspension was prepared by passing through a 100 µm cell strainer and samples were then counted for cell viability using Trypan blue (Sigma-Aldrich, Singapore). Later, cells were stained against CD3 (clone 500A2 BUV563; BD Biosciences), CD4 (clone RM4–5 BV650; BD Biosciences), CD8 (clone 53-6.7 BV510; BD Biosciences) and CD25 (clone PC61 BUV395; BD Biosciences). The FACS data was acquired on a BD FACSymphony and the instrument settings, such as detector voltage and fluorophore gain, were set up using murine spleen cell suspension. The flow data was analysed using FlowJo V10.5 software (FlowJo LLC) and expressed as % of cell population.

### 4.6. Statistical Analysis

Data were analysed using a non-parametric Kruskal–Wallis one-way ANOVA (GraphPad Prism V8.0.0, San Diego, CA, USA). *p* < 0.05 was considered statistically significant. Data are expressed as mean ± S.D. unless otherwise indicated.

## 5. Conclusions

To the best of our knowledge, this is the first study that reports the semi-automated synthesis of [^18^F]FB-IL2 using the commercially available Scintomics GRP™ module. We were able to increase the amount of available [^18^F]FB-IL-2 3.8-fold (i.e., 3.8 ± 0.7% RCY) and reduce the total synthesis time by ~30 min compared to previously developed methods (i.e., 1.0 ± 0.4% RCY). The improved synthesis of [^18^F]FB-IL-2 provides significantly greater end-of-synthesis yields that facilitate the distribution of the radiopharmaceutical to remote imaging centers without cyclotron facilities. Tracer properties including in vitro binding, in vivo tissue distribution and kinetics are comparable to earlier published data on [^18^F]FB-IL-2. PET imaging in MC38 and CT26 tumour-bearing mice indicates that [^18^F]FB-IL-2 can distinguish tumours with higher levels of CD25+ tumour infiltrating lymphocytes. 

## Data Availability

Not applicable.

## Supplementary Materials

Figure S1: Schematic diagram of Scintomics sequence visualization file of semi-automated [^18^F]SFB synthesis with positions of reagent vials, reaction vial, transfer lines and a syringe, Table S1. Scintomics GRP™ time control file (sequence) for semi-automated [^18^F]SFB, Table S2. Gradient elution profile for [^18^F]SFB analysis by RP-HPLC, Figure S2. Radio- and UV chromatograms of the DMSO reconstituted radiosynthon, [^18^F]SFB, Figure S3. Calibration curve of *N*-succinimidyl 4-fluorobenzoate, SFB, Table S3. Calibration curve calculations of N-succinimidyl 4- fluorobenzoate, SFB, Table S4. Gradient elution for [^18^F]FB-IL-2 analysis by RP-HPLC, Figure S4. Calibration curve of *N*-(4-fluorobenzoyl)-interleukin-2, FB-IL2, Table S5. Calibration curve calculations of *N*-(4-Fluorobenzoyl)-Interleukin-2, FB-IL2.
