# Supplementary material for: An Improved Synthesis of N-(4-[18F]Fluorobenzoyl)-Interleukin-2 for the Preclinical PET Imaging of Tumour-Infiltrating T-cells in CT26 and MC38 Colon Cancer Models
Source: Molecules. 2021 Mar 19;26(6):1728. doi: 10.3390/molecules26061728 (PMC8003786; doi:10.3390/molecules26061728)
Supplement: Supplementary file 1 [file molecules-26-01728-s001.pdf]

Supporting Information for

An Improved Synthesis of N-(4-[^{18}F]Fluorobenzoyl)-Interleukin-2 for the Preclinical PET Imaging of Tumour-Infiltrating T cells in CT26 & MC38 Colon Cancer Models

Shivashankar Khanapur [1,*], Fui Fong Yong [1], Siddesh V. Hartimath [1], Lingfan Jiang [1],Boominathan Ramasamy [1], Peter Cheng [1], Pradeep Narayanaswamy [2], Julian L. Goggi [1] and Edward George Robins [1,3,*]

[1] Singapore Bioimaging Consortium, Agency for Science, Technology and Research (A* STAR), 11 Biopolis Way, #01-02 Helios, Singapore, 138667

[2] Sciex, R&D, Blk 33, #04-06 Marsiling Ind Estate Road 3 Woodlands Central Indus. Estate, Singapore, 739256

[3] Clinical Imaging Research Centre, 14 Medical Drive, #B01-01 Centre for Translational Medicine, Yong Loo Lin School of Medicine, National University of Singapore, Singapore, 117599

* Correspondence:

shivashankar@sbic.a-star.edu.sg ; Tel.: +65 6478 7053

Edward_Robins@sbic.a-star.edu.sg ; Tel.: +65 6478 7001

Received: date; Accepted: date; Published: date

Contents

Detailed Scintomics GRP4V System Setup 3

Schematic diagram of Scintomics GRP™ visualization file 5

Time control Sequence file for semi-automated labeling 6

Molar Activities of N-succinimidyl 4- [18F]fluorobenzoate, [18F]SFB 9

Molar Activities of N-(4-[18F]Fluorobenzoyl)-Interleukin-2, [18F] FB-IL-2 11

Detailed Scintomics GRP4V System Setup

The Scintomics GRP4V module consists of a reactor unit, a master valve unit, radioactivity detector units (three gamma sensors), and a high-precision motor syringe with 20-port PEEK rotors for solvent and gas supply. Synthesis module was controlled by scintomics control centre (SCC) software and self-diagnosis system (ISDS) hardware interface. Three DMSO resistant cassettes were mounted on the PEEK rotors of GRP4V. Each automated valve unit was equipped with a stopcock-valve manifold with five 3-way valves (Scintomics). Manifolds and syringes were connected by standard silicone tubing according to supplementary Figure S1. The reaction vessel was a flat-bottomed 15 ml glass vial that was connected to modular valves through silicone tubing and a long needle for addition of reagents/ withdrawal of reaction mixture and a vent.

Cassette Installation:

Place the manifolds tightly onto the Scintomics GRP™ synthesiser starting from manifold 3 onto valve positions 11-15.

- Load and start «Prepare Load» file
- All valves rotate into position 3 (downwards)
- Place all chemicals, 20 ml syringe, vials and silicone tubings according to Figure S1 below.

At the indicated valve positions on the DMSO resistant cassettes the following was mounted (Figure S1):

Valve 1 (Horizontal)

Cut the silicone tube to required length and connect it to mass flow controller (MFC)

Valve 1 (Vertical)

Empty

Valve 2 (Vertical)

Male-to-male connector, attach 0.45 x 10 mm needle, remove the cover and mount eluent vial

(15 mg Kryptofix 222 and 3 mg K_2CO_3 in a mixture of 40 μL H_2O & 960 μL acetonitrile in 1.5 ml GC vial)

Valve 3 (Vertical)

For connection of V-Vial outlet (long tube deep inside V-vial touching bottom); insert tubing in activity sensor (γ1)

Valve 4 (Vertical)

Tubing with white male / male connectors, connect to pre-conditioned Light QMA cartridge and place cartridge into the radioactivity sensor holder (γ 2)

Valve 5 (Vertical)

Install blue spike; Mount 3 ml anhydrous Acetonitrile vial (Vial 1)

Valve 5 (Horizontal)

Tubing with white male / male connectors, connect to Valve 10 horizontal

Valve 6 (Vertical)

Tubing with white male / female connectors, connect to pre-conditioned Light QMA cartridge and place it into the radioactivity sensor holder (γ 2)

Valve 6 (Horizontal)

Tubing with white male / male connectors, connect to Valve 11 horizontal

Valve 7 (Vertical)

Tubing to reactor inlet

Valve 8 (Vertical)

Install spike and mount TSTU (20 mg) vial after dissolving in 1.5 ml acetonitrile

Valve 9 (Vertical)

Install spike; mount 20 μl 1M TPAOH in H_2O vial for deprotection

Valve 10 (Vertical)

Install spike; Mount precursor vial (5 mg) after dissolving precursor in 1 ml anhydrous DMSO

Valve 10 (Horizontal)

Tubing with white male / male connectors, connect to Valve 5 horizontal

Valve 11 (Vertical)

For connection of V-Vial inlet (white male connector)

Valve 12 (Vertical)

Tubing with orange male/white female connectors, connect to tubing from ^{18}O-water recovery bottle

Valve 13 (Vertical)

Tubing with white /white male connectors; tubing to final vial

Valve 14 (Vertical)

Connect 20 ml syringe and insert syringe into syringe pump

Valve 15 (Vertical)

Tubing to reactor outlet and place it into the radioactivity sensor holder (γ 3)

Valve 15 (Horizontal)

Tubing with green male/white female connectors, connect to tubing from waste bottle

Schematic diagram of Scintomics GRP™ visualization file

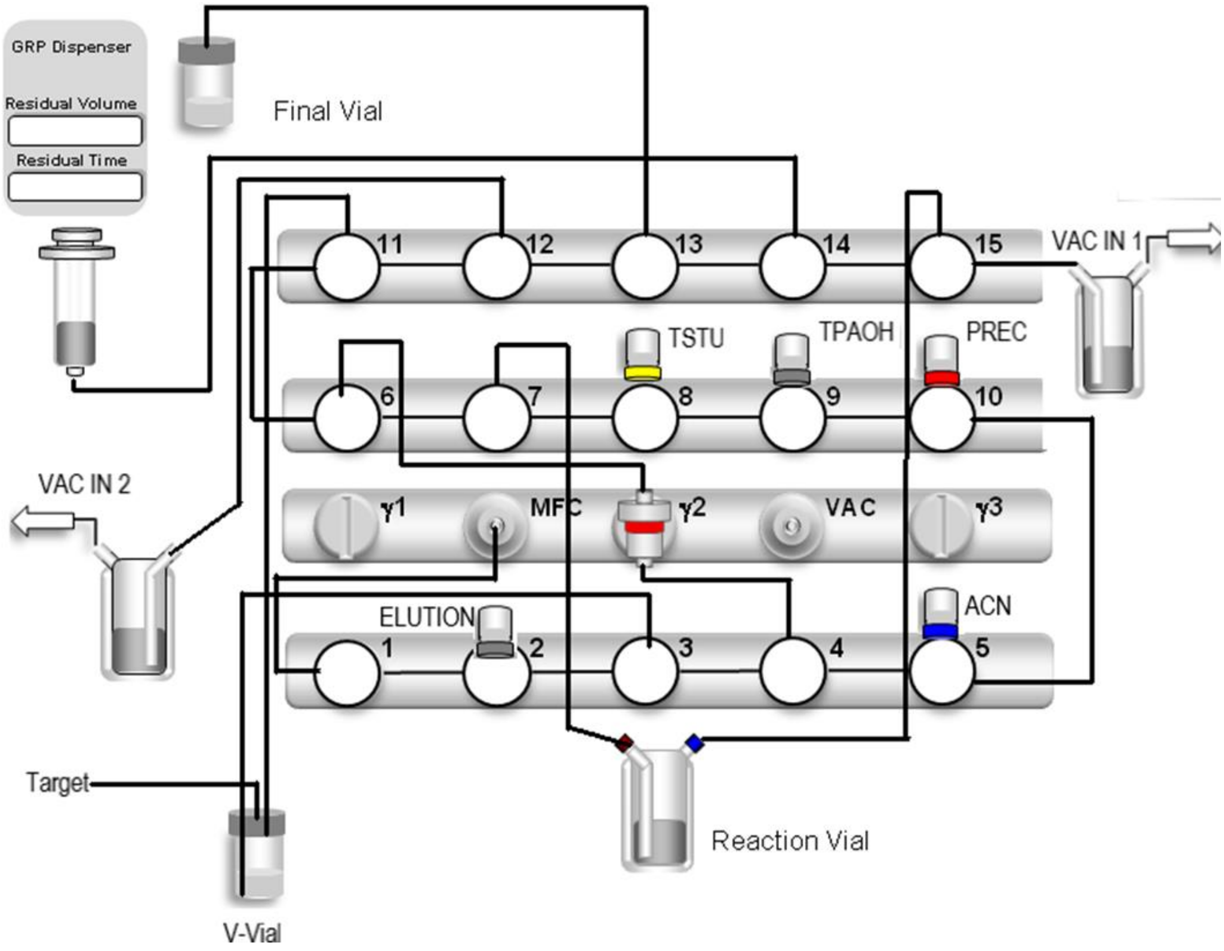

Figure S1. Schematic diagram of Scintomics GRP™ visualization file of semi-automated [^{18}F]SFB synthesis with positions of reagent vials, reaction vial, transfer lines and a syringe.

Time control Sequence file for semi-automated labeling

Table S1. Time control file (Sequence)

Time [min]	Function	Parameter
0.01	Analog Output	**Vacuum Pump = 5000 mV**
0.01	Valve Position	**Vac IN Switch = Vac IN1**
0.01	Temperature	**Temperature Zone 1 = 70 °C**
0.01	Start Chromatogram	**Ch. 4,5,6,7,8,9 (500 ms SliceWidth)**
0.05	Valve Position	**Valve 11 = Position 2**
0.05	Valve Position	**Valve 12 = Position 4**
0.05	Valve Position	**Vac IN Switch = Vac IN2**
0.06	Display Information	**"Fluoride ready? Please press continue!"**
0.10	Wait for Input Signal	**Virtual Switch 1 = ON**
0.14	Display Information	**"Trapping of Fluoride"**
0.15	Valve Position	**Valve 6 = Position 4**
0.15	Valve Position	**Valve 4 = Position 4**
0.15	Valve Position	**Valve 11 = Position 3**
0.18	Valve Position	**Valve 3 = Position 2**
0.48	Valve Position	**Valve 3 = Position 3**
0.48	Flowrate Flow	**40.00 ml**
0.58	Valve Position	**Valve 6 = Position 3**
0.58	Valve Position	**Valve 4 = Position 3**
0.58	Valve Position	**Valve 11 = Position 4**
0.59	Flowrate Flow	**200.00 ml**
0.64	Valve Position	**Valve 6 = Position 4**
0.64	Valve Position	**Valve 11 = Position 3**
0.64	Valve Position	**Valve 4 = Position 4**
0.64	Flowrate Flow	**0.00 ml**
0.67	Valve Position	**Valve 3 = Position 2**
0.94	Valve Position	**Valve 3 = Position 3**
0.94	Flowrate Flow	**40.00 ml**
1.09	Valve Position	**Valve 11 = Position 4**
1.09	Valve Position	**Valve 6 = Position 3**
1.09	Flowrate Flow	**200.00 ml**
1.09	Valve Position	**Valve 4 = Position 3**
1.14	Valve Position	**Valve 11 = Position 3**
1.14	Valve Position	**Valve 4 = Position 4**
1.14	Valve Position	**Valve 6 = Position 4**
1.14	Flowrate Flow	**0.00 ml**
1.17	Valve Position	**Valve 3 = Position 2**
1.52	Valve Position	**Valve 3 = Position 3**
1.67	Flowrate Flow	**40.00 ml**
1.83	**Temperature**	**Temperature Zone 1 = 95 °C**

Aqueous fluoride activity from cyclotron to V-vial of Scintomics (0.01–0.06 min)

Trapping of fluoride activity onto a pre-conditioned QMA-carbonate cartridge (0.14–1.83 min)

Time [min]	Function	Parameter	
1.83	Valve Position	**Valve 12 = Position 3**	
1.83	Flowrate Flow	**0.00 ml**	
1.84	Valve Position	**Vac IN Switch = Vac IN1**	
1.84	Display Information	**"Elution of Fluoride"**	
1.84	Valve Position	**Valve 7 = Position 4**	
1.84	Valve Position	**Valve 15 = Position 2**	
1.85	Valve Position	**Valve 6 = Position 3**	
1.86	Valve Position	**Valve 6 = Position 2**	
1.91	Valve Position	**Valve 2 = Position 2**	Elution of fluoride activity from QMA-carbonate cartridge to reactor vial
2.33	Flowrate Flow	**40.00 ml**	
2.34	Valve Position	**Valve 2 = Position 3**	
2.54	Valve Position	**Valve 4 = Position 3**	
2.54	Valve Position	**Valve 7 = Position 3**	
2.54	Valve Position	**Valve 6 = Position 3**	
2.55	Display Information	**"Azeotropic Drying of Fluoride"**	
2.55	Valve Position	**Valve 7 = Position 2**	
5.55	Display Information	**"Transfer of MeCN into Reactor"**	
5.57	Valve Position	**Valve 7 = Position 3**	
5.57	Valve Position	**Valve 6 = Position 4**	
5.58	Valve Position	**Valve 5 = Position 2**	Azeotropic drying of fluoride activity
5.62	Valve Position	**Valve 5 = Position 3**	
5.62	Valve Position	**Valve 7 = Position 2**	
5.62	Valve Position	**Valve 6 = Position 3**	
5.63	Display Information	**"Azeotropic Drying of Fluoride"**	
7.64	Flowrate Flow	**0.00 ml**	
7.65	Display Information	**"Transfer of Precursor"**	
7.65	Temperature	**Temperature Zone 1 = 110 °C**	
7.66	Analog Output	**Vacuum Pump = 0 mV**	
7.72	Valve Position	**Valve 15 = Position 4**	
7.73	Valve Position	**Valve 14 = Position 2**	
7.73	Valve Position	**Valve 10 = Position 4**	
7.74	Dispenser	**Dispenser 1 Aspirates 10000 µl from 1 (100000 µl/min)**	
7.82	Flowrate Flow	**100.00 ml**	Transfer of precursor & fluorination reaction
7.82	Valve Position	**Valve 10 = Position 3**	
8.12	Flowrate Flow	**0.00 ml**	
8.13	Display Information	**"Labeling; Fluorination reaction"**	
8.13	Valve Position	**Valve 7 = Position 3**	
8.13	Valve Position	**Valve 15 = Position 3**	
8.30	**Dispenser**	**Dispenser 1 Dispenses 10000 µl to 1 (142500 µl/min)**	

Time [min]	Function	Parameter
**15.87**	Temperature	**Temperature Zone 1 = 60 °C**
**19.48**	Temperature	**Temperature Zone 1 = 90 °C**
**19.49**	Display Information	**"Transfer 20uL 1M tetrapropylamonium hydroxide"**
**20.51**	Valve Position	**Valve 9 = Position 4**
**20.51**	Valve Position	**Valve 7 = Position 2**
**20.51**	Valve Position	**Valve 15 = Position 2**
**20.51**	Analog Output	**Vacuum Pump = 5000 mV**
**20.81**	Valve Position	**Valve 9 = Position 3**
**20.81**	Flowrate Flow	**40.00 ml**
**23.81**	Display Information	**"Additional azeotropic evaporation step"**
**23.83**	Display Information	**"Transfer of MeCN into reactor"**
**23.85**	Valve Position	**Valve 6 = Position 4**
**23.85**	Valve Position	**Valve 7 = Position 3**
**23.86**	Valve Position	**Valve 5 = Position 2**
**23.90**	Valve Position	**Valve 5 = Position 3**
**23.91**	Valve Position	**Valve 6 = Position 3**
**23.91**	Valve Position	**Valve 7 = Position 2**
**26.09**	Flowrate Flow	**0.00 ml**
**26.11**	Temperature	**Temperature Zone 1 = 60 °C**
**26.11**	Analog Output	**Vacuum Pump = 0 mV**
**27.66**	Temperature	**Temperature Zone 1 = 90 °C**
**27.66**	Display Information	**"Coupling reaction with TSTU"**
**27.72**	Valve Position	**Valve 15 = Position 4**
**27.72**	Valve Position	**Valve 14 = Position 2**
**28.73**	Valve Position	**Valve 15 = Position 2**
**28.73**	Analog Output	**Vacuum Pump = 5000 mV**
**28.74**	Valve Position	**Valve 7 = Position 2**
**28.76**	Valve Position	**Valve 8 = Position 4**
**28.89**	Flowrate Flow	**40.00 ml**
**28.91**	Analog Output	**Vacuum Pump = 0 mV**
**28.92**	Valve Position	**Valve 8 = Position 3**
**29.03**	Flowrate Flow	**0.00 ml**
**29.15**	Valve Position	**Valve 7 = Position 3**
**29.15**	Valve Position	**Valve 15 = Position 3**
**29.46**	Valve Position	**Valve 5 = Position 2**
**29.50**	Valve Position	**Valve 5 = Position 3**
**29.52**	Valve Position	**Valve 14 = Position 3**
**29.52**	Flowrate Flow	**100.00 ml**
**29.52**	**Analog Output**	**Vacuum Pump = 5000 mV**

Hydrolysis & azeotropic evaporation (20.51–26.11)

Coupling reaction with TSTU (27.66–29.52)

Time [min]	Function	Parameter
30.0	Analog Output	**Vacuum Pump = 0 mV**
30.0	Flowrate Flow	**0.00 ml**
32.4	Temperature	**Temperature Zone 1 = 60 °C**
35.6	Valve Position	**Valve 14 = Position 4**
35.7	Valve Position	**Valve 7 = Position 4**
35.7	Dispenser	**Dispenser 1 Aspirates 19000 µl from 1 (15000 µl/min)**
37.1	Valve Position	**Valve 13 = Position 2**
37.1	Valve Position	**Valve 15 = Position 3**
37.1	Dispenser	**Dispenser 1 Dispenses 19000 µl to 1 (100000 µl/min)**
37.4	Valve Position	**Valve 7 = Position 3**
37.4	Valve Position	**Valve 13 = Position 3**
37.4	Valve Position	**Valve 5 = Position 2**
37.4	Dispenser	**Dispenser 1 Aspirates 10000 µl from 1 (15000 µl/min)**
38.2	Valve Position	**Valve 7 = Position 4**
38.2	Valve Position	**Valve 5 = Position 3**
38.2	Dispenser	**Dispenser 1 Dispenses 10000 µl to 1 (100000 µl/min)**
38.4	Valve Position	**Valve 15 = Position 2**
38.4	Dispenser	**Dispenser 1 Aspirates 19000 µl from 1 (15000 µl/min)**
39.9	Valve Position	**Valve 13 = Position 2**
39.9	Valve Position	**Valve 15 = Position 3**
39.9	Valve Position	**Valve 7 = Position 3**
39.9	Dispenser	**Dispenser 1 Dispenses 19000 µl to 1 (100000 µl/min)**
40.2	Flowrate Flow	**100.00 ml**
40.2	Valve Position	**Valve 13 = Position 4**
40.5	Flowrate Flow	**0.00 ml**
40.6	Display Information	**"Crude [^{18}F]SFB is ready!"**
40.6	Valve Position	**Valve 13 = Position 3**
40.6	Valve Position	**Valve 14 = Position 3**
40.6	Valve Position	**Valve 7 = Position 3**
40.6	Valve Position	**Valve 5 = Position 3**
40.6	Valve Position	**Valve 15 = Position 3**
40.7	Stop Chromatogram	**Channel 4,5,6,7,8,9**
40.7	**Stop all**	**-**

Transfer of crude [^{18}F]SFB (steps 35.6–40.6)

Molar Activities of N-succinimidyl 4- [^{18}F]fluorobenzoate, [^{18}F]SFB

An aliquot (20 µL) of DMSO reconstituted [^{18}F]SFB was injected to analytical radio-HPLC and molar activity was calculated based on mass calibration curve.
Analytical HPLC conditions:
Column: Synergy Fusion-RP, 250 × 4.6mm, 4u, 100 Å
Mobile phase: A) 0.1%TFA in H_2O B) Acetonitrile
Flow rate: 1.5 mL/min, λ = 254 nm

Table S2. Gradient elution profile for [^{18}F]SFB analysis by RP-HPLC

Time (min)	Pump B Concentration in %
0.01	10
1	10
5	100
8	100
9	10
14.01	STOP

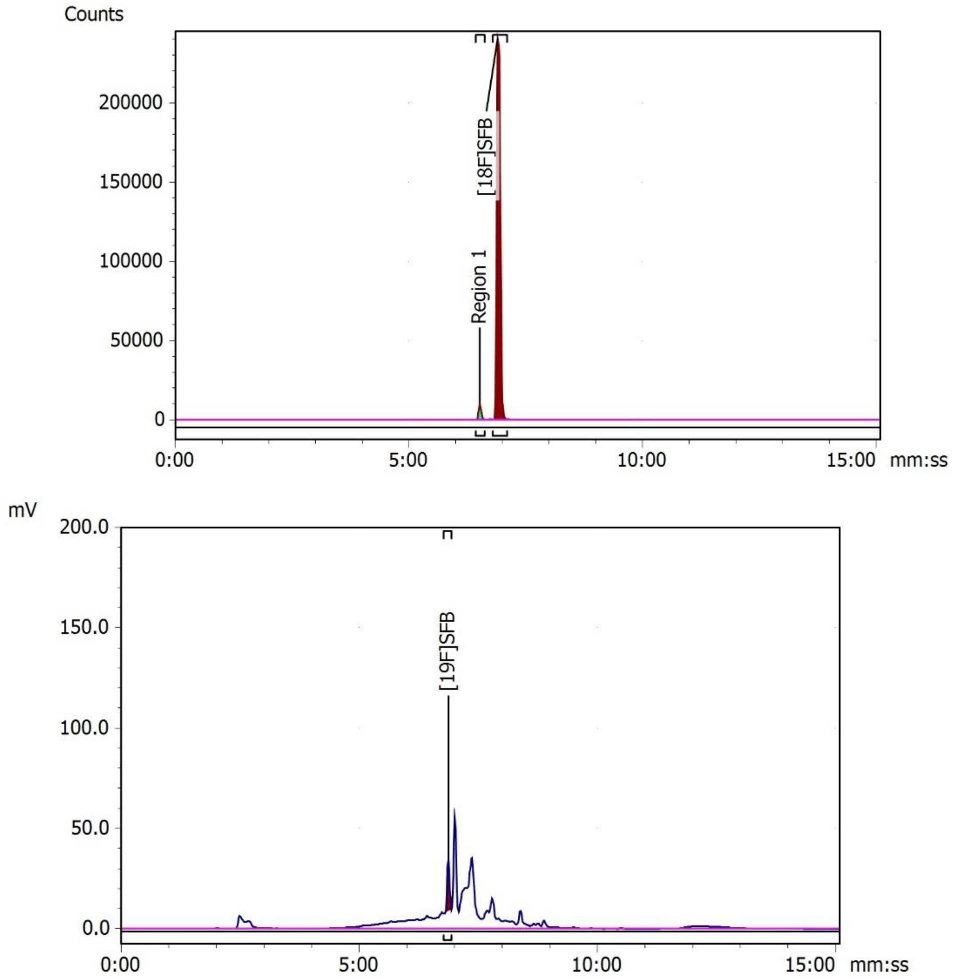

Figure S2. Radio- and UV chromatograms of the DMSO reconstituted radiosynthon, [^{18}F]SFB

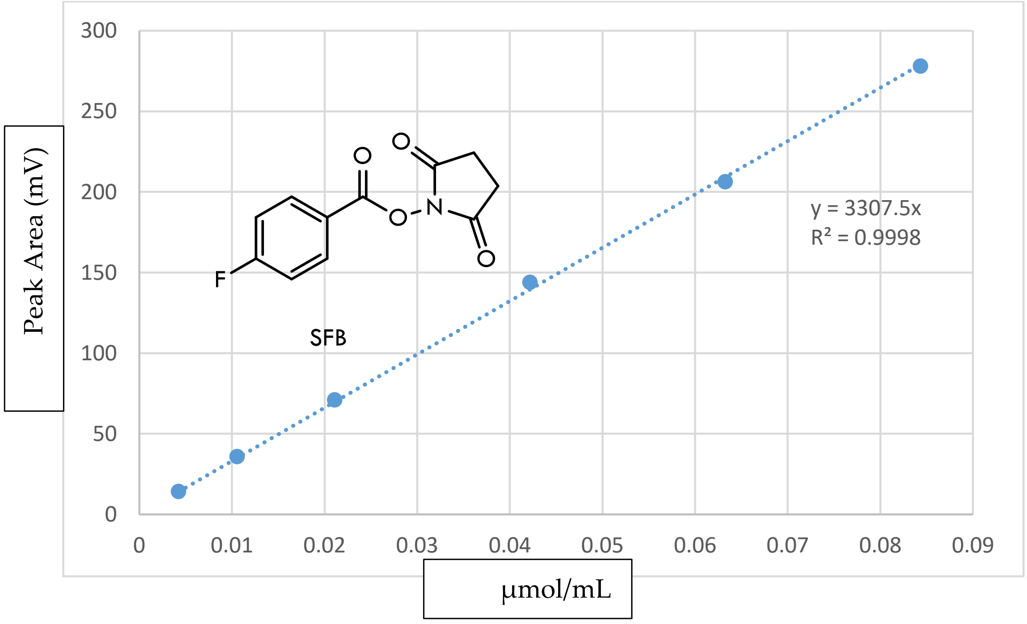

Figure S3. Calibration curve of N-succinimidyl 4- fluorobenzoate, SFB

Table S3. Calibration curve calculations of N-succinimidyl 4- fluorobenzoate, SFB

					Allowable range 85-115%
**S.No.**	**Concentration (µg/mL)**	**Concentration (µmol/mL)**	**Peak area (mV)**	**Observed concentration (µmol/mL)**	**Accurancy (%, µmol/mL)**
1	20	0.0843	278.2	0.0841	99.74
2	15	0.0632	206.4	0.0624	98.67
3	10	0.0421	144	0.0435	103.26
4	5	0.0210	71.1	0.0214	101.97
5	2.5	0.0105	36	0.0108	103.26
6	1	0.0042	14.4	0.0043	103.26

Molar Activities of N-(4-[^{18}F]Fluorobenzoyl)-Interleukin-2, [^{18}F] FB-IL-2

An aliquot (5 µL) of formulated [^{18}F]FB-IL2 was injected to analytical radio-HPLC and molar activity was calculated based on mass calibration curve.
Analytical HPLC conditions:
Column: Aeris Widepore C4, 3.6 µm, 150 mm × 2.1 mm, 200 Å
Mobile phase: A) 0.1%TFA in H_2O B) 0.1 % TFA in acetonitrile
Flow rate: 0.9 mL/min, λ = 280 nm
Table S4. Gradient elution for [^{18}F] FB-IL-2 analysis by RP-HPLC

Time (min)	Pump B Concentration in %
0.01	5
0.2	5
5	95
6	95
14	5
14.01	STOP

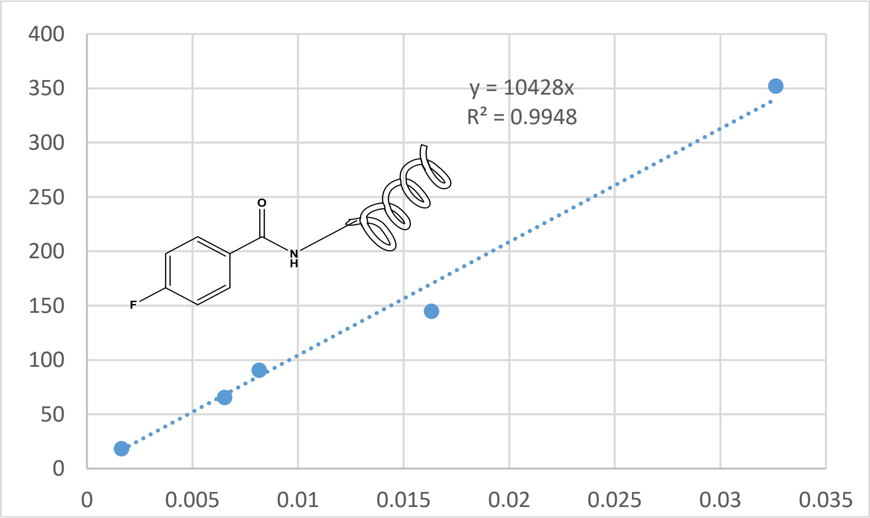

Figure S4. Calibration curve of N-(4-Fluorobenzoyl)-Interleukin-2, FB-IL2

Table S5. Calibration curve calculations of N-(4-Fluorobenzoyl)-Interleukin-2, FB-IL2

					Allowable range 85-115%
**S.No.**	**Concentration (µg/mL)**	**Concentration (µmol/mL)**	**Peak area (mV)**	**Observed concentration (µmol/mL)**	**Accurancy (%, µmol/mL)**
1	25	0.00163	18.1	0.00173	106.41
2	100	0.00652	65.4	0.00627	96.12
3	125	0.00815	90.3	0.00865	106.17
4	250	0.01631	144.8	0.01388	85.13
5	500	0.03262	352	0.03375	103.47
